# Change in brain network topology as a function of treatment response in schizophrenia: a longitudinal resting-state fMRI study using graph theory

**DOI:** 10.1038/npjschz.2016.14

**Published:** 2016-04-27

**Authors:** Jennifer Ann Hadley, Nina Vanessa Kraguljac, David Matthew White, Lawrence Ver Hoef, Janell Tabora, Adrienne Carol Lahti

**Affiliations:** 1 Department of Psychiatry and Behavioral Neurobiology, University of Alabama, Birmingham, AL, USA; 2 Department of Neurology, University of Alabama, Birmingham, AL, USA

## Abstract

A number of neuroimaging studies have provided evidence in support of the hypothesis that faulty interactions between spatially disparate brain regions underlie the pathophysiology of schizophrenia, but it remains unclear to what degree antipsychotic medications affect these. We hypothesized that the balance between functional integration and segregation of brain networks is impaired in unmedicated patients with schizophrenia, but that it can be partially restored by antipsychotic medications. We included 32 unmedicated patients with schizophrenia (SZ) and 32 matched healthy controls (HC) in this study. We obtained resting-state scans while unmedicated, and again after 6 weeks of treatment with risperidone to assess functional integration and functional segregation of brain networks using graph theoretical measures. Compared with HC, unmedicated SZ showed reduced global efficiency and increased clustering coefficients. This pattern of aberrant functional network integration and segregation was modulated with antipsychotic medications, but only in those who responded to treatment. Our work lends support to the concept of schizophrenia as a dysconnectivity syndrome, and suggests that faulty brain network topology in schizophrenia is modulated by antipsychotic medication as a function of treatment response.

## Introduction

Schizophrenia is a complex mental illness that manifests in different symptom dimensions. Options for pharmacological management are currently limited to dopamine D2 receptor blockers, which alleviate positive symptoms, but the success of treatment is variable.^1^ This may in part be attributable to our lack of a comprehensive understanding of the pathophysiology underlying the disorder and the mechanisms of action of antipsychotic medications on a systems level.

A number of structural and functional neuroimaging studies have provided evidence in support of the hypothesis that faulty interactions between spatially disparate brain regions are central to the disease.^2^ A common method to examine functional interactions between brain regions is resting-state connectivity, which leverages low-frequency fluctuations in the blood-oxygen-level-dependent (BOLD) signal to make inference on the brain’s functional organization.^3^ Most often, resting-state studies use independent components analysis or seed-based approaches to delineate functionally connected brain regions or networks of regions. More recently, graph theoretical methods have been applied to neuroimaging data in an attempt to obtain complementary measures on brain topology, perhaps reflecting brain organization principles on a more global level.^4^ In graph theory, the brain is conceptualized as a complex network of highly interconnected regions.^5^ It is defined by a set of nodes with edges between them, and structured in a way to optimize the interplay between segregation and integration of functionally specialized areas.^6^ Its topology, capturing the relations between nodes regardless of their physical location, can be described with various computations.^7^ Global efficiency, a measure of network integration, reflects the speed of information transfer across nodes, and the global clustering coefficient, a measure of network segregation, quantifies local interactions between nodes.^8^ Small-world properties in the overall brain network structure, i.e., highly clustered, yet globally interconnected nodes, have been reported in functional connectivity studies in healthy humans.^7,9,10^

Studies utilizing graph theoretical approaches have reported aberrant structural and functional brain network topology in schizophrenia. Although studies examining structural topology generally report increased network segregation (i.e., greater clustering) and decreased integration (i.e., lower global efficiency),^11^ studies investigating functional topology more commonly find reduced clustering and increased or unchanged global efficiency.^12–16^ Importantly, it is unclear to which extent discrepancies between structural and functional abnormalities reflect effects of antipsychotic medications, as all functional studies thus far enrolled subjects treated with antipsychotic medications at the time of scanning. There is preliminary evidence that dopamine antagonists may modulate global brain network topology. Administration of a single dose of sulpiride, a selective dopamine D2 receptor antagonist, resulted in reduced global efficiency in healthy human subjects.^7^ However, we do not know whether graph theoretical measures are sensitive to dopaminergic modulation in patients with schizophrenia, or whether they can be leveraged to advance our mechanistic understanding of antipsychotic drug action.

In this prospective, longitudinal study, we used resting-state fMRI (functional magnetic resonance imaging) to examine global network topology in patients with schizophrenia at two time points: (1) while unmedicated, and (2) after 6 weeks of treatment with risperidone. On the basis of the existing literature, we hypothesized that brain network topology, both clustering and global efficiency (two of the most commonly reported graph metrics), in unmedicated patients would be abnormal when compared with matched healthy controls. In an exploratory manner, we also examined if antipsychotic medication would partially restore network topology as a function of clinical response to treatment.

## Results

We observed no significant differences between patients and controls in age, sex, parental socioeconomic status, or smoking (Table 1). In subjects with Brief Psychiatric Rating Scale (BPRS) change scores available, 20 were responders, and 8 were non-responders. Baseline BPRS total scores as well as positive and negative symptom subscales did not differ between patients who eventually responded to treatment and those who did not (all *P*>0.20). Dose of risperidone at endpoint was numerically, and clinically meaningfully, higher in non-responders compared with responders (5.14±1.07 mg and 4.06±1.3 mg, respectively; *t*=−1.947; *P*=0.06).

### Small-world range of densities

**Figure 1:**
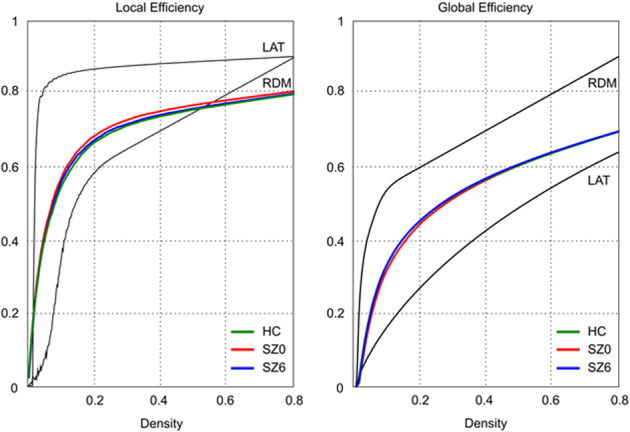
Small-world range of network densities. Local and global efficiency (*y* axis) as a function of density (*x* axis) for a random graph (RDM), a regular lattice (LAT), and participant brain networks. The small-world regime is defined as the range of densities 0.018⩽*K*⩽0.47 for which the global efficiency curve for the brain networks is greater than the global efficiency curve for the lattice and less than the global efficiency curve for the random graph. HC, healthy controls; LAT, regular lattice; RDM, Random graph; SZ, schizophrenia.

Efficiency increased as a function of cost in all networks (random, lattice, and investigated groups), where a random graph is characterized by a random, pattern-less series of connections between nodes, and a lattice graph by a uniform pattern of connections between all nodes. The random graph had higher global but lower local efficiency than the lattice graph. As expected, we found that the efficiency curves of brain networks were intermediate between the same parameters estimated in lattice and random graphs in healthy controls (HC) and schizophrenia (SZ) at both time points (Figure 1). We identified the small-world range of densities to be 0.018–0.468, at densities lower than 0.018 the graphs fragmented.

### Functional segregation

**Figure 2:**
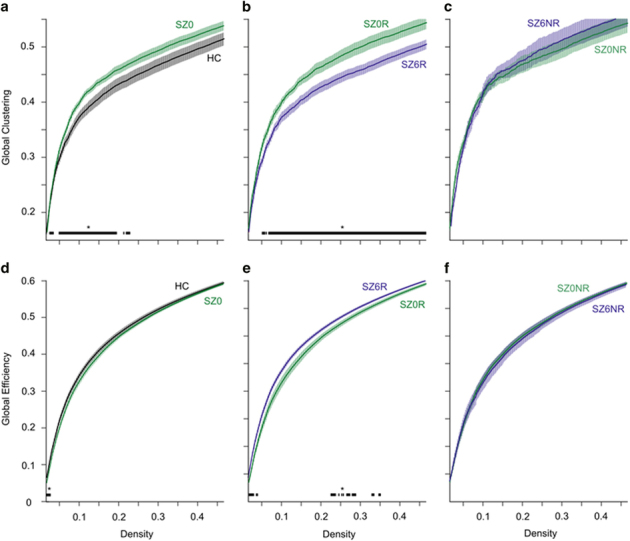
Brain network topology in healthy controls and patients with schizophrenia, and changes in patterns as a function of treatment response. Top row: global clustering coefficient across the small-world range. (**a**) Global clustering in healthy controls (HC) and unmedicated patients with schizophrenia (SZ0). (**b**) Global clustering in patients with good clinical response at baseline (SZ0R), and after 6 weeks of treatment (SZ6R). (**c**) Global clustering in patients with poor clinical response at baseline (SZ0NR), and after 6 weeks of treatment (SZ6NR). Bottom row: (**d**) global efficiency in HC and SZ0. (**e**) Global efficiency in patients with good clinical response at baseline, and after 6 weeks of treatment. (**f**) Global efficiency in patients with poor clinical response at baseline, and after 6 weeks of treatment. Lines represent the group mean, shaded regions correspond to the s.e.m. Starred regions indicate the range of densities where differences in global clustering and local efficiency are significant for the following comparisons (*P*<0.05). HC, healthy controls; SZ, schizophrenia.

In unmedicated patients with schizophrenia, we observed increased global clustering, predominantly at lower densities, when compared with healthy controls. Clustering did not differ between medication-naive and previously medicated patients. After 6 weeks of treatment, global clustering significantly decreased in the vast majority of densities across the small-world range in patients who responded well to antipsychotic medications, but not in those who did not have clinical improvement of symptoms (Figures 2a–c). To assess possible effects of motion, we entered mean framewise displacement as a covariate for analyses of group differences at a low-cost network threshold (*K*~0.1), similar to,^7^ and report significantly higher global clustering in unmedicated patients with schizophrenia compared with controls (F=5.07; *P*=0.03), which is consistent with our findings at this density when framewise displacement is not taken into account. We found a negative correlation between global clustering and BPRS total scores, but no relationships between global clustering and BPRS-positive or -negative symptom subscales.

### Functional integration

Global efficiency in unmedicated patients with schizophrenia was decreased in unmedicated patients compared with controls only at lower densities. Global efficiency did not differ between medication-naive and previously medicated patients. When we entered mean framewise displacement as a covariate for analyses of group differences at a low-cost network threshold (*K*~0.1), we report no significant group differences (F=0.65; *P*=0.43), which is consistent with our findings at this density when framewise displacement is not taken into account. Those patients who showed clinical improvement with treatment, showed an increase in global efficiency in the same density range, but also in some additional densities at the lower end of the spectrum. Patients who did not respond to antipsychotic medication had no changes in global efficiency after 6 weeks (Figure 2d–f). We found no relationships between global efficiency and BPRS-positive or -negative symptom subscales.

## Discussion

Here we examined brain network topology in unmedicated patients with schizophrenia and explored the effects of antipsychotic medications in a longitudinal study design applying graph theoretical measures to resting-state fMRI data. When compared to healthy controls, unmedicated patients demonstrated topology abnormalities within the small world range. These topological abnormalities are modulated over six weeks of antipsychotic therapy only in individuals responding to treatment.

We identified small-world properties of brain network topology in both groups, which is consistent with reports across different types of neuroimaging data, and suggestive of a highly conserved general principle of connectome organization.^6^ Within the small-world range, we found lower global efficiency as well as greater global clustering coefficients in unmedicated patients compared to healthy controls predominantly in the more sparsely connected network densities. In contrast, several previous studies reported increased global efficiency or decreased clustering coefficients that were interpreted as ‘subtle randomization’ of brain networks in schizophrenia.^14,17,18^ To our knowledge, all previously published studies examining functional brain network topology using graph theoretical approaches have included medicated subjects only, which may explain discrepancies in findings. Examining structural topology with diffusion tensor imaging, Zhang *et al*.^19^ reported evidence of disruptions in topology, specifically between frontal, parietal, and subcortical regions, despite preserved small worldness in medication-naive first-episode schizophrenia patients. Similarly, and consistent with our results, Zalesky *et al*.^20^ found evidence of decreased efficiency and increased clustering in structural topology in a large group of medicated patients with schizophrenia.

In this study, we report exploratory evidence that brain network topology may be sensitive to dopamine D2 receptor blockage, observing a decrease in the global clustering coefficient across the entire small-world range and an increase in global efficiency at lower densities, at least in those patients who clinically benefitted from medication. This is consistent with Rubinov *et al*.^21^ who found a correlation between clustering coefficient in electroencephalogram with antipsychotic dose, and was interpreted as medication exerting a ‘normalizing influence’. Taken together, it appears that brain network topology abnormalities, specifically decreased global clustering observed in medicated patients, may at least in part be attributable to medication effects rather than subtle randomization of networks intrinsic to the illness.

Several studies using more traditional ways to analyze resting-state data have also reported that antipsychotic medications may modulate functional connectivity. Our group has reported a partial attenuation of aberrant connectivity between the ventral tegmental area, a region rich of dopaminergic projections, and the thalamus after 1 week of treatment with risperidone, and attenuated dysconnectivity in the dorsal attention network after 6 weeks of treatment in patients with schizophrenia.^22,23^ Furthermore, two recent studies reported that resting-state connectivity between the hippocampus and caudate was modulated by antipsychotic medication as a function of clinical response.^24,25^

There are several plausible explanations as to the biological underpinnings of observed changes in brain network topology in relationship to drug effects. Oscillations in the gamma frequency range are believed to be generated by γ-Aminobutyric-acid interneurons and to synchronize brain activity. There is evidence of *N*-methyl-d-aspartate receptor hypofunction on these neurons,^26^ likely resulting in glutamatergic excess,^27–29^ in the pathophysiology of schizophrenia. It is conceivable that antipsychotic medication may alleviate aberrant synchronization of gamma oscillations through reduction of glutamate,^28,30,31^ and restore more normal patterns of functional topology of the brain. Alternatively, observed changes in functional topology may reflect changes in integrity of white matter structures. Diffusion tensor imaging studies have identified widespread aberrant structural connectivity abnormalities^32^ that may be affected by antipsychotic medication.^33^

Our findings have to be considered in context of several strengths and limitations. To minimize variance in the data, we only enrolled subjects that had no exposure to antipsychotic medications for at least 10 days preceding the baseline scan, used a single antipsychotic medication to treat psychosis, and carefully matched groups on several factors including parental socioeconomic status and smoking. Resting-state data are prone to contamination of non-neuronal signals.^34^ We applied rigorous preprocessing and motion correction to our data to minimize head motion artifacts,^35,36^ and conducted wavelet analysis to diminish the effect of slowly decaying positive auto correlations taking into account the long-memory properties of the BOLD signal.^7^ Because there is no generally accepted way to identify an optimal threshold for graph construction to apply to a data set, we decided to threshold the association matrix at different values to create a range of binary adjacency matrices, in an effort to describe network properties as a function of changing connection density.^7,37^ This allows characterization of the network across the small-world range, but comes at the cost of increasing the possibility of statistical type I errors. Because known effective treatments cannot be withheld from patients, we did not have a placebo group in our study, rendering it impossible to definitively attribute changes in network topology to effects of medication rather than time alone. Also, daily dose of risperidone at the second scan differed between groups, with non-responders being treated with clinically meaningfully higher doses of medication. However, this does not come as a surprise, as dose titration was based on clinical response.

In summary, our findings suggest a disturbance in network topology on the scale of the whole brain in schizophrenia that is modulated by antipsychotic medication as a function of clinical response to treatment. Future studies investigating structural and neurochemical correlates of treatment response in relationship to functional network topology will allow us to advance our mechanistic understanding of antipsychotic drug action.

## Materials and methods

Unmedicated patients with schizophrenia or schizoaffective disorder (SZ) were recruited from those who sought treatment at the University of Alabama at Birmingham (UAB) and matched HC relatives, matched on age, sex, parental occupation, and smoking were recruited from the university’s newspaper and flyers. Approval for this study was obtained by the UAB Institutional Review Board. Written informed consent to participate in the study was obtained after subjects were deemed competent to provide consent.^38^

Exclusion criteria were medical or neurological disorders, use of medications known to affect brain function, pregnancy, moderate to severe substance use disorders (after the exception of nicotine) within six months of imaging as defined by DSM 5,^39^ history of loss of consciousness, and MRI contraindications. HC were also free from current or lifetime psychotic disorders in themselves and first-degree relatives, as assessed with the Diagnostic Interview for Genetic Studies.^40^

Diagnosis was established using the Diagnostic Interview for Genetic Studies, subjects’ medical records, and consensus by two board certified psychiatrists (A.C.L. and N.V.K.). Symptom severity was assessed weekly using the Brief Psychiatric Rating Scale.^41^ All SZ were off antipsychotic medication for at least ten days; medication was not discontinued to meet this criterion. Patients received a 6-week trial of risperidone. Medication management was done by A.C.L. and N.V.K.; dosing was based on therapeutic and side effects. Starting doses were 1–3 mg; titration was done in 1- to 2-mg increments. Concomitant antidepressant or mood stabilizing medication was allowed to be used as indicated (Twelve subjects received benztropine, two trazodone, and one each got mirtazapine, amitriptyline, and valproic acid). Compliance was monitored by pill counts at each visit. Response to risperidone was defined as decrease of BPRS total scores of 30% or more between baseline and week-6 assessment. BPRS scores from week five were carried forward for three subjects to calculate response.

A total of 32 SZ subjects and 32 HC completed resting-state scans at baseline. Seven SZ dropped prior to the second scan, one SZ scan was excluded due to excessive head motion during scanning at the second scan, and resting-state scans were not obtained for two SZ subjects at the second time point; leaving 22 SZ subjects in analysis for week 6.

### MRI acquisition

All imaging was performed on a 3-T head-only scanner (Magnetom Allegra, Siemens Medical Solutions, Erlangen, Germany), equipped with a circularly polarized transmit/receive head coil. Resting-state fMRI scans were acquired during a 5-min gradient recalled echo-planar imaging sequence (TR/TE=2,000/30 ms, flip angle=70°, field of view=192×192 mm^2^, 64×64 matrix, 6-mm slice thickness, 1-mm gap, 30 axial slices). During the scan, participants were instructed to keep their eyes open and stare passively ahead. High-resolution structural scans were acquired using the three-dimensional T1-weighted magnetization prepared rapid acquisition gradient-echo sequence (TR/TE/inversion time (TI)=2300/3.93/1100 ms, flip angle=12°, 256×256 matrix, 1-mm isotropic voxels). All MRI scans were reviewed for abnormalities by a neuroradiologist.

### Data preprocessing

Using the statistical parametric mapping package SPM8 (Wellcome Trust Centre for Neuroimaging, London, UK), resting-state data were preprocessed as previously described.^22,42^ Briefly, data were slice-timing corrected, realigned, co-registered, normalized to Montreal Neurologic Institute space using DARTEL,^43^ and spatially smoothed. To remove physiological noise, a nuisance regression using the six motion parameters and their first derivatives as regressors and step-wise data ‘scrubbing’ eliminating severely motion contaminated time points were performed^34,36^ (proportion of frames scrubbed in HC was 16.4%, in SZ at baseline was 19.2%, and in SZ at week 6 was 19.5%). Then, a principle component analysis was used to extract white matter and cerebral spinal fluid components necessary to explain 90% of signal variance from those regions. These extracted components were used as regressors in a second nuisance regression.^44^ No global signal regression was performed. To assess the group differences in motion and changes in motion over time, we calculated mean framewise displacements, and found no difference in motion between groups (HC: 0.17±0.12 mm; SZ: 0.22±0.23 mm; d.f.=62; *t*=−1.17; *P*=0.24), or over time (baseline: 0.25±0.28 mm; week 6: 0.24±0.23 mm; d.f.=19; *P*=0.51).

### Connectivity matrices and graph theory measures

**Figure 3:**
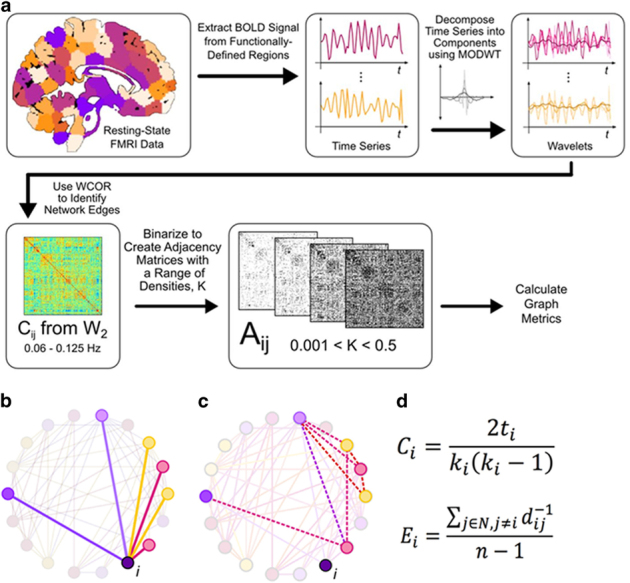
(**a**) Graph theory analysis pipeline. Clustering coefficient, a measure of network segregation. (**b**) For the region *i*, the solid lines shown are the *edges* connecting it to other regions. The total number of these edges is that node’s degree, *k*_*i*_. (**c**) The set of edges that connect *i*’s neighbors to each other, shown as dashed lines, is defined as *t*_*i*_. (**d**) *C*_*i*_, the clustering coefficient of each node *i* is the ratio of actual connections to possible connections between a node’s neighbors. It is computed for each node and averaged to give the global clustering coefficient. Global efficiency, a measure of network integration. (**b**) Node *i* shown with the edges connecting it to its neighbors (solid lines). Path length, *d*_*ij*_, is the number of edges between nodes *i* and *j*. Distance, *d*_*ij*_^*−1*^, is the inverse of path length. (**d**) *E*_*i*_, *efficiency* of node *i*, is the average distance to all other nodes in the network. Global efficiency, the average efficiency of all nodes in a network, reflects the speed of information transfer among nodes of a network or, network integration. *A_ij_*, adjacency matrix; BOLD, blood oxygen level dependent; *C_ij_*, correlation matrix; *K*, density; MODWT, maximal overlap discrete wavelet transform; *W*_2_, wavelet scale two; WCOR, wavelet correlation.

Mean time series were extracted for each subject from 278 regions of interest defined by the Shen *et al.*^45^ functional atlas. The frequency band of interest was identified by applying the maximal overlap discrete wavelet transform to each time series using the WMTSA toolbox for MATLAB^46^ and selecting wavelet scale 2, which corresponds approximately to the frequency range of interest 0.03–0.06 Hz. For this wavelet scale, wavelet correlations between signals in the 278 anatomical regions were calculated. This resulted in a 278-node weighted, undirected graph (abbreviated *C*_*ij*_) for each subject (Figure 3). The graph theory methods used in this study, implemented with the brain connectivity toolbox,^47^ are most accurate when binary, undirected graphs are used as inputs, as discussed by Achard and Bullmore.^7^ We therefore choose to evaluate an arbitrary range of network thresholds to convert *C*_*ij*_ to corresponding adjacency matrices *A*_*ij*_. There is no clear consensus on which thresholds are most appropriate, but it is accepted that the brain functions in the small-world range of densities.^7^ We applied a range of thresholds to produce all possible densities, then evaluated the resulting small worldness of the adjacency matrices and choose thresholds that would produce valid graphs in the small-world range. For each participant’s graphs, corresponding to the range of densities examined (0.018–0.468), we evaluated measures of functional segregation and integration. In other words, we examined the same global network measures at changing thresholds to summarize network metrics across the small-world range. Statistical group differences in functional connectivity were assessed using two-tailed two-sample *t*-tests on the groups’ network parameter for each density, and are reported as significant for *P*<0.05. In an exploratory fashion, we also assessed changes in connectivity over time in SZ using paired-sample *t*-tests.

## Figures and Tables

**Table 1:** Demographic characteristics and clinical measures^a^

	*Patients (*n*=32)*	*Controls (*n*=32)*	*t/χ*^b^	P*-value*
*Demographic characteristics*				
Age (years)	33.60 (10.38)	34.03 (10.61)	0.17	0.86
Sex (male/female)	23/9	18/14	1.70	0.30
Parental SES^b^	7.00 (6.27)	7.06 (4.50)	0.05	0.96
Smoking status (Y/N)	27/5	21/11	3.00	0.15
Smoking (packs per day)	0.70 (0.53)	0.70 (0.65)	−0.05	0.96
				
*Diagnosis*				
Schizophrenia	29	—		
Schizoaffective disorder	3	—		
				
*Illness characteristics*				
Illness duration (years)	10.69 (9.90)	—		
First episode	10	—		
				
*Prior antipsychotic treatment*				
Antipsychotic naïve	15	—		
Antipsychotic-free interval (months)	22.99 (44.46)	—		
				
*BPRS baseline (n=32)*				
Total score	47.41 (10.37)	—		
Positive symptom subscale	9.59 (3.17)	—		
Negative symptom subscale	6.72 (2.68)	—		
				
*BPRS; week 6 (n*=*25)*				
Total score	30.00 (8.71)	—		
Positive symptom subscale	4.68 (2.43	—		
Negative symptom subscale	5.28 (2.51)	—		

Abbreviations: BPRS, Brief Psychiatric Rating Scale; N, no; SES, socioeconomic status; Y, yes.

a Mean (s.d.) are shown unless indicated otherwise.

b Ranks determined from the Diagnostic Interview for Genetic Studies, reported on 1–18 scale; higher rank (lower numerical value) corresponds to higher socioeconomic status. Data were unavailable for two patients.
